# *In silico* Analysis of *CHD4* Mutations Reveals Domain‐Specific Impacts on Cardiovascular Disorders Among Patients With Rare Diseases

**DOI:** 10.1155/humu/3575977

**Published:** 2026-02-25

**Authors:** Apolonia Novillo, Marta Ysbert, Rocío Brea, Alicia María Hidalgo-Estévez, Fadoua El Abdellaoui-Soussi, Pablo Gómez-del Arco

**Affiliations:** ^1^ Department of Cell Biology and Histology, Faculty of Medicine, Complutense University of Madrid, Madrid, Spain, ucm.es; ^2^ Human Genetic Variability Group, Hospital La Paz Institute for Health Research–IdiPAZ (La Paz University Hospital–Universidad Autónoma de Madrid–Getafe University Hospital–Universidad Europea de Madrid), Madrid, Spain; ^3^ Institute for Rare Diseases Research, Instituto de Salud Carlos III (ISCIII), Madrid, Spain, isciii.es; ^4^ Department of Basic Health Sciences, Universidad Rey Juan Carlos, Alcorcón, Spain, urjc.es; ^5^ Center for Stem Cells and Organoid Medicine (CuSTOM), Cincinnati Children′s Hospital Medical Center, Cincinnati, Ohio, USA, cincinnatichildrens.org; ^6^ Centro de Investigación Biomédica en Red de Enfermedades Cardiovasculares (CIBERCV), Madrid, Spain

**Keywords:** cardiovascular disease, CHD4 mutation, congenital heart disease (CHD), Sifrim–Hitz–Weiss syndrome

## Abstract

Chromodomain‐helicase‐DNA‐binding protein 4 (CHD4) is a critical ATP‐dependent chromatin remodeler that plays fundamental roles in transcriptional repression, DNA damage repair, and lineage specification, making it indispensable for cardiovascular development and function. Pathogenic *CHD4* mutations are linked to syndromic and nonsyndromic conditions, often presenting with severe cardiac and vascular anomalies. However, most of these mutations are unique and nonrecurrent, complicating variant classification. In this study, we establish a connection between recent advances in CHD4 structure and function and 36 pathogenic *CHD4* mutations associated with rare diseases, including Sifrim–Hitz–Weiss syndrome, moyamoya angiopathy, and childhood idiopathic epilepsy with sinus arrhythmia, all of which exhibited cardiomyopathy, congenital heart defects, and/or vascular abnormalities. Among these mutations, 33 were missense variants, one was an in‐frame small insertion, one, an in‐frame small deletion, and one, a splice‐site variant. Variants were classified according to the ACMG guidelines and subsequent refinements, integrating clinical, functional, population, and *in silico* (REVEL‐based PP3/BP4) evidence, and cross‐referenced with the ClinVar database to prioritize candidates for further association and functional studies. We classified the missense variants as follows: seven as pathogenic (P), nineteen as likely pathogenic (LP), one as likely benign (LB), and six as variants of uncertain significance (VUS). The splice‐site variant was predicted to cause nonsense‐mediated decay and reduced CHD4 expression, whereas the structural variants were predicted to exert moderate effects on protein function. LP/P variants associated with congenital heart defects were significantly enriched within the ATPase/helicase domain (*p* = 0.027), suggesting impairing ATPase motor activity. Nevertheless, several severe heart malformations, including tetralogy of Fallot were linked to pathogenic or LP variants, such as C467Y (plant homeodomain [PHD]), M202I (high‐mobility group [HMG]), and Y1345D (C‐terminal domain). In contrast, other variants located in the N‐ and C‐terminal regions were more often associated with vascular phenotypes, suggesting domain‐specific roles of CHD4 in cardiovascular disease. These findings establish CHD4 as a key regulator of cardiovascular pathophysiology, though a clear genotype–phenotype correlation remains elusive. Further functional validation is essential to elucidate CHD4′s molecular mechanisms, aiding in diagnostic and therapeutic developments.

## 1. Introduction

Cardiovascular diseases (CVDs) remain the leading cause of global mortality, accounting for 31% of deaths worldwide [1]. Familial hypercholesterolemia, various types of cardiomyopathies, thoracic/aortic aneurysms, congenital heart diseases (CHDs), coronary artery diseases, heart failure, and strokes are progressive pathologies linked to CVDs [2]. Although environmental and lifestyle factors play critical roles in CVD development, genetic predisposition is increasingly recognized as a key determinant in disease susceptibility, progression, and patient‐specific treatment responses [3–5].

CHDs, the most common congenital abnormalities in newborns, arise from disruptions during cardiac embryogenesis, affecting structures such as the myocardium, cardiac valves, septa, and associated vasculature. Over 350 genes have been implicated in CHDs, yet more than 55% of cases remain genetically unexplained [6–8]. *De novo* mutations (DNMs) in chromatin‐regulating genes, including *CHD4* (chromodomain helicase DNA‐binding protein 4), account for approximately 8% of syndromic CHD cases, often presenting alongside neurodevelopmental abnormalities [9, 10]. These mutations frequently result in protein loss‐of‐function, exhibiting incomplete penetrance [11]. Such diseases often arise from complex genetic and environmental interactions, though the underlying causative mechanisms remain elusive [12].

CHD4, the core component of the nucleosome remodeling and deacetylase (NuRD) complex and the CHD4‐ADNP‐HP1 (ChAHP) complex, is a critical regulator of cardiovascular biology. In mice, Chd4 orchestrates chromatin structure and gene expression during both embryonic and postnatal development [13, 14]. Conditional knockout (KO) models have demonstrated its indispensability for cardiac development; embryonic depletion of *Chd4* is deleterious. In adults, the function of Chd4 is conserved, with its deletion in the heart leading to dilated cardiomyopathy and arrhythmias due to aberrant overexpression of skeletal muscle sarcomeric proteins [13, 14]. Similarly, *Chd4*′s deletion in terminally differentiated skeletal muscle results in the upregulation of cardiac sarcomeric proteins, culminating in myopathy [13]. These findings underscore the vital role of CHD4 in maintaining proper‐striated muscle identity by controlling gene expression.

Beyond its role in cardiac development, Chd4 is indispensable for vascular integrity. It regulates extracellular matrix (ECM) deposition around blood vessels, forming a critical biomechanical barrier against blood flow pressures [15–17]. Conditional deletion of *Chd4* in endothelial cells leads to vascular rupture, edema, and embryonic lethality, partly due to dysregulation of Wnt signaling pathways [15, 17]. Additionally, Chd4 is necessary for the proper development of lymphatic and liver sinusoidal endothelial cells, with endothelial *Chd4* KO resulting in edema and malformed lymphatic vessels [15]. These findings highlight the pivotal role of CHD4 in vascular and endothelial system homeostasis.

Heterozygous mutations in the *CHD4* gene caused a neurodevelopmental disorder in humans (Sifrim–Hitz–Weiss syndrome [SIHIWES]; OMIM: #617159) with variable congenital defects affecting other systems, including heart defects and skeletal abnormalities [18–20]. These mutations often result in protein loss‐of‐function, impacting critical processes during cardiac development and vascular homeostasis. Furthermore, Chd4 interacts with transcription factors such as Nkx2.5 and Gata4 during embryogenesis and Znf219 in adulthood, which recruit Chd4 to regulate gene expression programs in the myocardium [21, 22]. The diversity of CHD4 mutations across its domains suggests that each region or domain of the protein is physiologically relevant, contributing to a wide spectrum of human pathologies.

Recent studies have uncovered a spectrum of mutations in the *CHD4* gene, ranging from single nucleotide polymorphisms (SNPs) to structural variations, each potentially altering CHD4′s function. Structural studies using cryo‐electron microscopy (cryo‐EM) have provided insights into CHD4′s interaction with nucleosomes and the impact of specific disease‐associated mutations [23]. Complementary experimental models, including *Drosophila* systems, humanized mouse models, and CRISPR‐mediated gene editing in human embryonic stem cells, have been pivotal in elucidating the functional consequences of *CHD4* variants [24–26]. Furthermore, genome‐wide association studies (GWAS) have also identified correlations between *CHD4* variants and cardiovascular anomalies, offering population‐level insights into its role [27]. Integrating genomic data with bioinformatic tools has enabled the prediction of variant pathogenicity and prioritized candidates for functional studies [6, 26, 28, 29]. These combined approaches enhance our understanding of how CHD4 mutations contribute to disease pathogenesis and suggest new avenues for therapeutic exploration.

Over the past decade, the vast diversity of *CHD4* mutations across multiple domains has underscored the physiological relevance of each region in CHD4‐associated human pathologies [23]. Functional mutations of distinct CHD4 domains likely contribute to the heterogeneity of CVDs observed in affected patients. In this study, we integrated structural, functional, and genetic data on *CHD4*, systematically compiling the spectrum of variants identified in patients with cardiovascular anomalies. By applying the ACMG guidelines and subsequent refinements, we assessed the pathogenic potential of these mutations, cross‐referencing our findings with the ClinVar database to prioritize variants for further association and functional studies. This approach is aimed at providing further information about the mechanisms by which CHD4 dysfunction contributes to cardiac and vascular disease. This research not only advances our understanding of CHD4‐related diseases but also lays the groundwork for potential diagnostic biomarkers and therapeutic interventions.

## 2. Material and Methods

### 2.1. Selection and Identification of *CHD4* Variants in Patients With CVD

We conducted a search for genetic variants associated with cardiovascular anomalies by analyzing published data on patients with distinct pathological phenotypes. Specifically, our investigation focused on three groups: (i) individuals diagnosed with SIHIWES (OMIM: #617159), a rare autosomal dominant intellectual developmental disorder characterized by macrocephaly, cryptorchidism, and a broad spectrum of congenital heart, urogenital, and skeletal anomalies [18–20, 30]; (ii) four patients presenting with moyamoya angiopathy (MMA; OMIM: % 252350), a rare cerebrovascular disease characterized by progressive bilateral stenosis of the distal internal carotid arteries leading to strokes starting in childhood through the fourth decade of life resulting in a range of neurologic deficits [29]; and (iii) individuals from four unrelated families with a rare childhood idiopathic epilepsy (IP) and sinus arrhythmia, for which four new mutations of *CHD4* were identified in eight patients [31].

Genetic variants of *CHD4* were searched using the following keywords: CHD4 and heart, CHD4 and CVD in PubMed, targeting research articles describing variants in patients with the disorders indicated above and with any cardiovascular anomaly. We manually identified 34 patients (22 SIHIWES, four MMA, and eight with a rare childhood IP) with cardiovascular anomalies carrying *CHD4* variants. In a first step, variants were searched in the ClinVar database (https://www.ncbi.nlm.nih.gov/clinvar/). Variants with allele frequency of > 0.001 in gnomAD exomes v4.1 (https://gnomad.broadinstitute.org/) were filtered out. In a second step, using several bioinformatic approaches an impact functional prediction was calculated. In a third step, the variants were classified according to recommendations from the American College of Medical Genetics and Genomics and the Association of Molecular Pathology (ACMG/AMP), taking into consideration PP3 criteria to support a variant being pathogenic and BP4 to support a variant being benign [32, 33].

### 2.2. *In silico* Prediction of *CHD4* Variants

The pathogenic effects of *CHD4* missense variants (Q14839) described in this study were predicted using several online bioinformatic tools such as PolyPhen‐2 (Polymorphism phenotype v2, http://genetics.bwh.harvard.edu/pph2) that analyzes the potential effect of amino acid substitution on the function and structure of protein and based on the probabilistic score it provides the result as benign, possibly damaging and probably damaging [34]; AlphaMissense, categorize “missense” mutations in different proteins as either “likely pathogenic”, “likely benign” or “uncertain”, producing a score that estimates the likelihood of a variant being pathogenic [35]. Furthermore, the effects of harmful variants on protein structure were examined using the HOPE server (https://www3.cmbi.umcn.nl/hope/, visited July 2023); the output providing results regarding amino acid properties, structure analysis, physical contacts, evolutionary conservation, and domain affectation [36]. As recommended by Pejaver et al. [33], we used REVEL, an ensemble method for predicting the pathogenicity of rare missense variants. Precomputed REVEL scores for *CHD4* missense variants were obtained from Ioannidis et al. [37] and applied as supporting evidence for variant classification. Specifically, these computational scores were used to support pathogenicity (criterion PP3) or benignity (criterion BP4), in accordance with ACMG criteria.

The interpretation of the output of the data from the different software tools was as follows: for HOPE as MetaRNN, scores closer to 1 (≥ 0.85) indicate a high likelihood of pathogenicity. For PolyPhen‐2, scores ≥ 0.978 support pathogenicity (PP3), whereas lower scores (≤ 0.113) suggest a benign effect (BP4) [33]. For AlphaFold, high confidence scores (≥ 0.9) typically indicate structural or functional disruption, implying pathogenicity. For REVEL, scores ≥ 0.644 support pathogenicity (PP3), whereas lower scores (≤ 0.290) suggest benignity (BP4). PolyPhen‐2 and REVEL may also yield scores that fall into an undetermined range and, as noted by Pejaver et al. [33], such values should not be used as supporting evidence (PP3/BP4) for variant classification. Additionally, *in silico* predictions for the three non‐missense variants in our study (one splice‐site variant, one small insertion, and one small deletion) were generated using the Ensembl Variant Effect Predictor (VEP) [38].

Variant classification was performed according to the 2015 ACMG/AMP guidelines and their subsequent refinements [32], integrating multiple lines of evidence. In addition to using *in silico* predictions as supporting evidence (PP3 and BP4), based on REVEL scores as recommended [33], we also incorporated functional evidence (PS3) from previous published experimental studies and model systems. Population frequency data (PM2) were evaluated using gnomAD exomes v4.1 database, and absence or extremely low allele frequency was considered as supportive of pathogenicity. Evidence from segregation and *de novo* occurrence (PS2) or assumed (PM6) was also applied where availability was granted. Each variant was classified as pathogenic, likely pathogenic, benign, likely benign, or of uncertain significance (VUS) based on the combination and strength of the evidence available (see Tables 1 and S2). Genetic variants commonly identified as pathogenic or likely pathogenic by ACMG classification were considered high‐risk SNPs. The prediction for each variant was compared to established classifications based on experimental and clinical data deposited in ClinVar.

**Table 1:** *In silico* predictions with HOPE, PolyPhen‐2(PoP‐2), AlphaMissense (AF) and REVEL for the 33 missense mutations, and ACMG classification of the *CHD4* variants considered in this study. NP, not available data in ClinVar database; VUS, variant of uncertain significance; CS, conflicting classification; LP, likely pathogenic; P, pathogenic; PP3 and BP4 criteria of pathogenicity and benignity, respectively.

**Mutation**	**ClinVar**	**HOPE/PoP-2/AF/REVEL scores**	**REVEL**	**ACMG classification (criteria)**	**Comparison (ACMG vs. ClinVAr)**
p.P8S	LP	0.24/0.91/0.09/0.294	Undetermined	VUS (PM2 + PM6)	Downgraded
p.M202I	VUS	0.90/0.98/1.0/0.788	PP3_moderate	Pathogenic (PS2 + PS3 + PM2 + PP3)	Upgraded
p.P286A	NP	0.16/0.02/0.05/0.239	BP4_supporting	VUS (PM2 + PP2 + BP4)	First classification
p.C467Y	NP	0.99/1.0/1.0/0.968	PP3_strong	Pathogenic (PS2 + PS3 + PM2 + PP3)	First classification
p.T494M	VUS	0.41/0.707/0.10/0.14	BP4_moderate	Likely benign (PM2 + BS2 + BP4)	Downgraded
p.K533E	VUS	0.23/0.35/0.44/0.316	Undetermined	Likely pathogenic (PS2 + PM2)	Upgraded
p.K810N	NP	0.66/1.0/1.0/0.548	Undetermined	Likely pathogenic (PS2 + PM2)	First classification
p.S851Y	P	0.98/0.99/1.0/0.985	PP3_strong	Likely pathogenic (PS2 + PM2 + PP3)	Downgraded
p. R887W	P	0.89/1.0/0.95/0.884	PP3_moderate	Likely pathogenic (PS2 + PM2 + PP3)	Downgraded
p.M954I	P	0.88/0.90/0.99/0.905	PP3_moderate	Likely pathogenic (PS2 + PM2 + PP3)	Downgraded
p.M954V	NP	0.91/1.0/0.82/0.938	PP3_strong	Pathogenic (PS2 + PS3 + PM2 + PP3)	First classification
p.M966K	LP	0.86/0.98/0.99/0.889	PP3_moderate	Likely pathogenic (PS2 + PM2 + PP3)	Unchanged
p.R992Q	CS	0.88/1.0/0.72/0.685	PP3_supporting	Pathogenic (PS2 + PS3 + PM2 + PP3)	Upgraded
p.G1003D	NP	0.89/1.0/1.0/0.785	PP3_moderate	Pathogenic (PS2 + PS3 + PM2 + PP3)	First classification
p.N1020S	LP	0.94/1.0/0.73/0.855	PP3_moderate	Likely pathogenic (PS2 + PM2 + PP3)	Unchanged
p.R1068H	P	0.91/1.0/0.97/0.933	PP3_strong	Pathogenic (PS2 + PS3 + PM2 + PP3)	Unchanged
p. E1094K	P	0.93/1.0/1.0/0.958	PP3_strong	Pathogenic (PS2 + PS3 + PM2 + PP3)	Unchanged
p.D1147E	LP	0.82/0.16/0.99/0.845	PP3_moderate	Likely pathogenic (PS2 + PM2 + PP3)	Unchanged
p.W1148L	P	0.92/0.511/1.0/0.76	PP3_supporting	Likely pathogenic (PS2 + PM2 + PP3)	Downgraded
p.A1178V	LP	0.55/0.06/0.91/0.462	Undetermined	Likely pathogenic (PS2 + PM2)	Unchanged
p.R1183H	P/LP	0.85/0.99/0.97/0.71	PP3_supporting	Likely pathogenic (PS2 + PM2 + PP3)	Unchanged
p.R1183C	P/LP	0.89/1.0/0.99/0.877	PP3_moderate	Likely pathogenic (PS2 + PM2 + PP3)	Unchanged
p.A1188V	NP	0.89/0.24/0.97/0.828	PP3_moderate	Likely pathogenic (PS2 + PM2 + PP3)	First classification
p.M1192R	NP	0.80/0.01/0.99/0.797	PP3_moderate	Likely pathogenic (PS2 + PM2 + PP3)	First classification
p.Y1249D	LP	0.90/0.40/1.0/0.856	PP3_moderate	Likely pathogenic (PS2 + PM2 + PP3)	Unchanged
p.Y1345D	NP	0.91/1.0/1.0/0.809	PP3_moderate	Likely pathogenic (PS2 + PM2 + PP3)	First classification
p.R1419H	LP	0.91/0.26/0.992/0.861	PP3_moderate	Likely pathogenic (PM2 + PM6 + PP2 + PP3)	Unchanged
p. V1608I	CS	0.07/0.01/0.07/0.171	BP4_moderate	Likely pathogenic (PS2 + PM2 + BP4)	Upgraded
p.E1646K	NP	0.26/0.455/0.15/0.459	Undetermined	Likely pathogenic (PS2 + PM2)	First classification
p.D1659E	NP	0.08/0.00/0.09/0.321	Undetermined	VUS (PM2 + PP2)	First classification
p.I1741V	LP	0.58/0.25/0.66/0.5	Undetermined	VUS (PM2 + PM6)	Downgraded
p.Y1758C	CS	0.90/0.98/0.97/0.734	PP3_supporting	VUS (PM2 + PM6 + PP3)	Unchanged
p.P1880S	VUS	0.87/0.997/0.89/0.773	PP3_moderate	VUS (PM2 + PM6 + PP3)	Unchanged

### 2.3. Update of CHD4 Protein Structure and Function

A bibliographic search was conducted using the keywords CHD4, structure, and the names of individual domains in PubMed to gather the most recent information on structural features of CHD4 protein (Q14839). A total of 12 publications were selected to comprehensively describe the structure of the protein, including its domains, their respective locations, methods of discovery, and associated functions.

### 2.4. Statistical Analysis

Associations between mutation location (ATPase/Helicase region vs. other domains) and the presence of heart anomalies in patients carrying *CHD4* mutations were evaluated using a two‐sided Fisher′s exact test, with significance defined as *p* < 0.05. Odds ratios and 95% confidence intervals were calculated to estimate strength of association using GraphPad Prism.

## 3. Results and Discussion

### 3.1. Update on CHD4 Protein Structure

The CHD4 protein is a member of the CHD Subfamily II, involved in ATP‐dependent chromatin remodeling. CHD4, a large protein of 1912 amino acids, functions as the ATP‐dependent helicase subunit of the NuRD complex, orchestrating nucleosome assembly and histone modifications to regulate gene expression. Understanding its structure has advanced significantly since its discovery in 1995 by [39, 40] in studies of dermatomyositis connective tissue [23, 41].

The structure of CHD4 represents a pivotal advance in understanding its role in chromatin remodeling. Early investigations primarily focused on individual domains of CHD4 using nuclear magnetic resonance (NMR) spectroscopy, which provided foundational insights into its structural features [42–44]. More recently, advances in X‐ray crystallography and cryo‐EM have enabled detailed visualization of CHD4′s spatial organization and its functional domains. These high‐resolution experimental methods have collectively facilitated the development of a comprehensive structural model of the protein [23, 45, 46]. Complementing these experimental approaches, computational modeling has played a critical role. The RCSB Protein Data Bank now hosts 13 experimentally derived models that delineate specific domains and structural elements of CHD4 [47]. Meanwhile, AlphaFold has produced a full‐length predictive model of CHD4, leveraging artificial intelligence to infer structural details entirely computationally (available in UniProt). Notably, AlphaFold also integrates functional insights by offering an AlphaMissense Pathogenicity Heatmap, which categorizes predicted missense mutations as likely benign, uncertain, or likely pathogenic [35] (Figure 1).

Figure 1(a) Structure of chromodomain helicase DNA binding protein 4 (CHD4). CHD4 consists of 1912 amino acid. The N‐terminal region (Residues 1–354) includes two intrinsically disordered regions (IDR 1/2), a high‐mobility group (HMG), and one aggregation prone region (APR). The central region (Residues 354–1230) contains two plant homeodomains (PHD 1/2), two chromodomains (CD 1/2) and the ATPase motor, compromising the ATPase/helicase domain and helicase 2 domain. The C‐terminal (Residues 1230–1912) includes C‐terminal Domain 1 (CTD1), a SANT‐SLIDE domain, an additional APR, and C‐terminal Domain 2 (CTD2). (b) Table summarizing CHD4 domains, residues coordinates, the methods used to describe the domains, and their molecular function of CHD4. References: [23, 42–46, 48–53].

**Figure:**
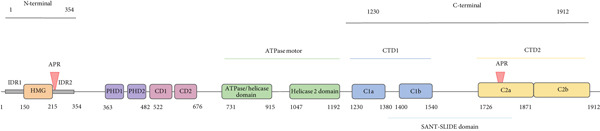
(a)

**Figure:**
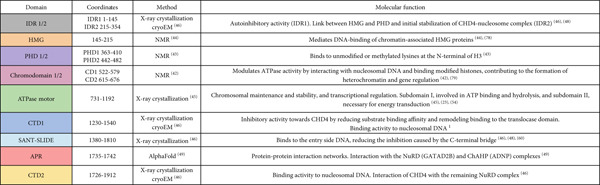
(b)

These advancements collectively reveal that CHD4 is a multifunctional protein organized into five major regions: the N‐terminal, plant homeodomain (PHD), double chromodomain (CD), ATPase motor, and C‐terminal regions. Each of these regions contributes uniquely to understanding the biological activity of CHD4, underscoring the importance of both experimental and computational methods in unraveling the functional complexity of this chromatin remodeler (Figure 1). Each CHD4 domain is described below in terms of its location, the method by which its structure was first determined, its function, and any additional information currently available.

### 3.2. N‐Terminal Region

The N‐terminal region (Residues 1–354), demonstrated to be essential for full remodeling activity, encompasses two intrinsically disordered regions (IDRs 1 and 2), a high‐mobility group (HMG) box‐like domain, and an aggregation prone region (APR) (Figure 1) [46]. The HMG‐like domain (82 amino acids, Figure 1) is helical and binds the DNA backbone, enhancing the remodeling properties of CHD4, although its specific role in remodeling remains incompletely understood [44].

Moreover, Zhong et al. [46] refined the structural roles of the IDRs in chromatin remodeling and protein interactions. IDRs 1 and 2 are highly charged, predicted to lack a defined tertiary structure, and significantly influence CHD4 activity. IDR1 exerts mild autoinhibition, whereas IDR2 facilitates the stabilization of the CHD4‐nucleosome complex. Mutational analysis of IDR1 identified a cluster of charged lysine residues that support nucleosome remodeling. Interestingly, truncating the N‐terminal region to exclude IDR1 slightly enhanced remodeling activity, suggesting modulatory elements in this region, though the mechanism is unclear. In IDR2, the amino acid composition, particularly the higher density of positively charged residues, plays a crucial role in nucleosome remodeling efficacy. Sequence scrambling in IDR2 did not significantly affect remodeling, but reducing negatively charged residues increased nucleosome affinity. Conversely, reducing positively charged residues substantially decreased affinity. Microscale thermophoresis experiments confirmed the importance of these charged residues in stabilizing the CHD4‐nucleosome complex. These findings suggest that IDR2, through its basic residues, actively participates in nucleosome remodeling by stabilizing the initial CHD4‐nucleosome interaction. The combined roles of the HMG‐like domain and IDRs underscore the importance of both structured and disordered regions in CHD4′s remodeling activity (Figure 1) [46, 48]. Recently, Tabar et al. identified the APR as instrumental in erythrocyte gene transcription, emphasizing its developmental relevance [49] (Figure 1).

### 3.3. Plant Homeodomain (PHD)

Central to the protein, the PHD (1/2, Residues 363–410 and 442–482) are zinc finger motifs that recognize histone modifications. Mansfield et al. [43] demonstrated their ability to bind unmodified and methylated lysines, which is critical for nucleosome recognition and interaction with histone H3. This domain mediates CHD4′s specificity in chromatin targeting and is characterized by conserved Cys‐His‐Cys modules containing two zinc ions; implicated in binding to histone H3 in their amino acid terminal tails (Figure 1) [43, 54].

### 3.4. Double Chromodomain (CD)

The CD (1/2, Residues 522–579 and 615–676) forms a highly conserved structural motif with significant roles in ATPase modulation and chromatin binding (Figure 1). Structurally it is folded in three‐stranded antiparallel *α*‐helixes and *β*‐sheets. The duplication of the CD in the CHD families confers to them the ability to have affinity to specific histone methylation patterns. In studies performed with dMi‐2, the deletion of CDs leads to the lack of nucleosome binding and mobilization. Thus, these domains display DNA and nucleosome binding activities [55–57]. Originally characterized in HP1 proteins, this domain stabilizes the interaction of CHD4 with nucleosomal DNA at superhelical location (SHL) +2 and participates in histone modification recognition. These properties underline its contribution to heterochromatin formation and gene repression [42].

### 3.5. ATPase Motor

The ATPase motor (ATPase Residues 731–915 and helicase Residues 1047–1192), shared by all CHD proteins, plays a central role in nuclear processes including transcriptional regulation and chromosomal maintenance. It contains seven conserved motifs arranged into two subdomains: Subdomain I, which mediates ATP binding and hydrolysis, and Subdomain II, which facilitates energy transduction [45, 50]. Systematic mutational analyses have shown that each motif is essential for ATPase/helicase activity [58].

X‐ray crystallography by Dürr et al. [45] revealed the structural basis of these subdomains and their roles in nucleosome repositioning. This domain underlies the “twist defect” model, a defining feature of CHD4′s ATPase‐driven chromatin remodeling mechanism [45, 46, 50] (Figure 1).

### 3.6. C‐Terminal Region

The C‐terminal region (Residues 1230–1912) includes two distinct domains, CTD1 and CTD2, further subdivided into functional subregions (e.g., C1a, C1b, C2a, and C2b). CTD1 has an inhibitory role, whereas CTD2 is implicated in nucleosome interaction and NuRD complex stabilization (Figure 1). In early work, these two domains were named as of unknown function (DUF1/2) [59], and several authors indicate that this region may possess a repressive transcriptional activity, since CHD4 and/or CHD3 interact with numerous corepressors (hunchback, NAB2, and RFP among others) through these modules [60–62]. Recently, Zhong et al. [46] have described that the C‐terminal region contains a regulatory module that includes an autoinhibitory motif. This motif interacts with the helicase domain, which is essential for the remodeling activity of CHD4, and it is important to point out that mutations here could lead to loss of regulation, resulting in aberrant CHD4 activity. Autoinhibition is relieved by previous unrecognized C‐terminal SANT‐SLIDE Domain split by approximately 150 residues of disordered sequence, most likely by binding of this domain to substrate DNA. In more detail, this domain contains two ordered regions (C1: 1200–1539 and C2: 1700–1912) split by around 160 residues that are predicted to be disordered (1310–1400 and 1540–1699). This specific range of residues includes the SANT‐SLIDE domain that forms a bipartite structure capable of binding double‐stranded DNA. This binding is essential for the chromatin remodeling activity of CHD4, allowing it to interact with nucleosome DNA effectively and preventing premature or inappropriate remodeling. The presence of these residues enhances the protein′s ability to remodel chromatin by promoting interactions with nucleosome DNA. Within the SANT‐SLIDE domain, there is a ~20‐residue loop dominated by charged, small, and polar residues. This loop is important for the dynamic behavior of the domain, which is necessary for its function in recognizing and binding to DNA. The SANT‐SLIDE domain exhibits a prominent basic surface flanked by acidic regions, which is indicative of its ability to interact with the negatively charged DNA. This electrostatic property is crucial for the affinity and specificity of DNA binding [46].

Up to now, references indicated that no DNA binding activity is linked to this domain; however, several authors show that the C‐terminal C2 region (Residues 1700–1912) is required for connecting CHD4 with the nucleosome remodeling and deacetylase (NuRD) complex [57]. Additionally, an APR (Residues 1735–1742) in CTD2 has been linked to essential interactions with transcription factors within the NuRD and ChAHP complexes, advancing our understanding of the regulatory network of CHD4. Furthermore, the C‐terminal domain is interspersed with disorder segments, which may play a role in the flexibility and adaptability of CHD4 during its interactions with nucleosomes, suggesting that they could be important for the dynamic nature of the remodeling activity of CHD4 [46, 48, 51].

### 3.7. *CHD4* Genetic Variants Spectrum in CVD

In this report we compile the information available in the literature describing 36 variants in the *CHD4* gene from different human pathological phenotypes: (i) SIHIWES (autosomal dominant intellectual disorder, OMIM: #617159); (ii) MMA (OMIM: %252350); and (iii) childhood IP with sinus arrhythmia; and using bioinformatic approaches we predicted the functional impact of these mutations in protein functionality. Significant variation in pathology across *CHD4* variants (see Table S1) suggests that *CHD4* may be a candidate disease gene implicated in a wide range of CVDs, despite the lack of an established genotype–phenotype correlation. The current information is highly valuable for characterizing expected phenotypes, as all patients in this study share various heart and cardiovascular anomalies (Table S1). These findings are of particular interest to the scientific community, given the emerging role of CHD4 in cardiac development and vascular integrity.

**Figure 2:**
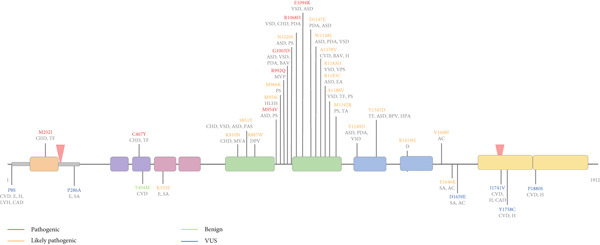
Localization of missense mutations of interest within the domains in chromodomain helicase DNA binding protein 4 (CHD4). The identifications of the mutations are in red (pathogenic), orange (likely pathogenic), blue (variant of uncertain significance), or green (benign or likely benign) according to the ACMG classification conducted in this study. In addition, the diseases (black) for each mutation are shown in the figure. The diseases shown are cerebrovascular disease (CVD), epilepsy (E), hypertension (H), left ventricular hypertrophy (LVH), coronary artery disease (CAD), congenital heart disease (CHD), tetralogy of Fallot (TF), sinus arrhythmia (SA), mitral valve anomaly (MVA), ventricular septal defect (VSD), atrial septal defect (ASD), pulmonary artery stenosis (PAS), dysplastic pulmonary valve (DPV), hypoplastic left heart syndrome (HLHS), pulmonary stenosis (PS), mitral valve prolapse (MVP), patent ductus arteriosus (PDA), bicuspid aortic valve (BAV), valvular pulmonar stenosis (VPS), Ebstein′s anomaly (EA), truncus arteriosus (TA), bicuspid pulmonary valve (BPV), hypoplastic pulmonary artery (HPA), dextrocardia (D), and aortic coarctation (AC) [9, 18–20, 26, 28, 29, 31, 63–65].

These 36 mutations, classified as in‐frame small insertion (1), in‐frame small deletion (1), splicing donor (1), and missense mutations (33) occurred in different locations across the whole gene. Variants influencing cardiovascular and cardiac phenotypes were found in the N‐terminal domain, the CHD, both lobes of the ATPase motor, and the CTD1/2 (Figure 2). Most of the missense mutations (79%) map to the ATPase motor and the C‐terminal domain (Table S2, Figure 2). It is of interest that some of *de novo* missense mutations in *CHD4* described here are also associated with other human diseases such as cancer as described in COSMIC database (see Table S1), and have also been described by other authors [23]. This overlap underscores the functional significance of the affected regions, particularly in processes such as chromatin remodeling, transcriptional regulation, and DNA repair. Furthermore, some patterns have been pointed out by some authors; the missense mutations located in the regions from SNF2 super family domain to DUF1087 domain, which is located between the ATPase motor and CTD1 domains (Residues 810–1345) were associated with Multisystem Developmental Disorders (MSDDs), whereas epilepsy‐related mutations were outside of this area [31]. This observation implies that mutations between the SNF2 superfamily domain and the DUF1087 domain may disrupt interactions or activities essential for normal developmental processes, whereas epilepsy‐associated mutations may impair distinct functional aspects of CHD4 or act through alternative molecular pathways. Further investigation into these domains and their respective mutations could provide valuable insights into the molecular mechanisms underlying these diverse phenotypes.

The association of mutations spanning the SNF2 superfamily to the DUF1087 domain with MSDDs suggests that this segment is crucial for interactions or functions necessary for proper development. However, the presence of some mutations in cancer highlights that dysfunction of CHD4 can manifest in various ways depending on the cellular and pathological context. For instance, developmental disorders may arise from impaired tissue‐specific developmental programs, whereas cancer may result from broader disruptions in genome stability or cell cycle regulation.

**Figure 3:**
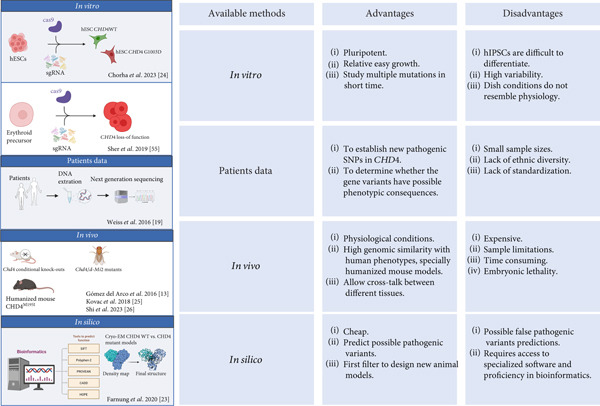
Overview of approaches used to study *CHD4* mutations, highlighting the advantages and limitations of each method. Figure created with BioRender.

This observation suggests that although some patterns of mutations point to domain‐specific phenotypic associations, the ultimate impact of *CHD4* mutations likely depends on the interplay between the specific mutation, the functional domain affected, and the broader cellular environment. Further studies, especially functional assays and *in vivo* models will be essential to elucidate the molecular mechanisms that distinguish these diverse phenotypes (Figure 3).

Mutated CHD4 residues are highly conserved—both evolutionarily, when comparing human CHD4 with orthologs in other vertebrates and even *Drosophila*, and within the CHD family itself—as previously reported [25, 26]. This conservation suggests that mutations affecting these residues are likely to disrupt CHD4 function and contribute to the patients′ phenotypes.

Interestingly, this analysis shows that more than 90% of the mutations described in *CHD4* are DNMs and are enriched in patients with neurodevelopmental disorders, SIHIWES syndrome, and CHD (Table S1). This agrees with the low‐sibling recurrence ratio (less than 3%) of patients with CHD [66, 67], the amount of DNMs of *CHD4* and its close relationship with syndromic heart disease [68] and the numerous studies have shown a direct relationship between neuronal impairment and CHD [69]. Moreover, it has been proven that the genetic mutations that cause neuronal disorders overlap with DNMs that cause CHD [69]. Their detection represents a strategy needed to link gene mutation with genotype and clinical aspects of patients. Furthermore, to assess whether the location of the variants within the protein influences the occurrence of heart anomalies, we compared mutations in the ATPase/helicase region (Variants 8–27; Table S1) with those located in other domains (Variants 1–7 and 28–36). Among the ATPase/helicase variants, 19 out of 20 were associated with reported heart anomalies, whereas only nine of 16 variants in other domains showed similar findings. A two‐sided Fisher′s exact test showed significant association between variant location and the presence of heart anomalies (*p* = 0.027), suggesting that mutations in the ATPase/helicase region are more likely to be associated with cardiac (heart) abnormalities compared with mutations in other protein domains (*O* *d* *d* *s* *r* *a* *t* *i* *o* = 12.67; 95*%*CI = 1.32–121.47). Overall, more quantitative studies are needed for the identification of co‐occurring and mutually exclusive *CHD4* mutations according to the specific pathology to identify patients that could potentially be targeted with directed therapies.

### 3.8. *In silico* Prediction of CHD4 Missense Variants

Analysis of computational predictions of the 33 *CHD4* missense variants using four *in silico* prediction tools (MetaRNN‐HOPE software, PolyPhen‐2, AlphaFold, and REVEL) revealed a moderate degree of predictive agreement for most variants (Table 1). Applying predefined thresholds, MetaRNN and AlphaFold classified 63.6% of variants as deleterious, whereas REVEL supported pathogenicity for 69.7% and benignity for 9.1% of variants, with 21.2% undetermined. PolyPhen‐2 showed a more dispersed profile, with 51.5% pathogenic, 15.2% benign and 33.3% undetermined calls. Overall, full concordance among all four tools was observed for 42.4% (14/33) of variants, whereas 57.6% showed at least one discordant prediction. PolyPhen‐2 contributes more divergent or undetermined outputs. For example, variants such as p.A1178V, p.A1188V, and p.M1192R, PolyPhen‐2 predictions were clearly discordant with the other *in silico* predictions. This is consistent with the known limitations and stricter calibrated thresholds of PolyPhen‐2 [33]. Thus, variants were interpreted based on available information compiled and classified according to the ACMG guidelines using *in silico* predictions (PP3 and BP4) derived from REVEL scores (Table 1). The results were compared with established classifications based on experimental and clinical data deposited in the ClinVar database (Table 1). A total of 33 *CHD4* missense variants were analyzed by comparing their reported ClinVar classifications with the integrated ACMG/AMP classifications proposed in this study. Overall, 36.5% (12/33) of variants remained concordant with ClinVar, whereas 27% (9/33) were newly classified (previously unannotated or with conflicting data), and 36.5% (12/33) were classified into a different category. Most discrepancies represented upgrades in pathogenicity confidence. Among variants originally annotated as VUS or CS, 2 were classified as likely pathogenic or pathogenic (2) after integrating multiple lines of evidence, including computational predictions, population frequency, segregation data, and functional studies. Conversely, one variant (T494M) showed downgrades toward likely benign or remained VUS after reevaluation (Y1758C and P1880S). This evaluation demonstrates that integrated ACMG/AMP interpretation provides improved resolution over existing ClinVar annotations.

### 3.9. Genetic Variants in the N‐Terminal Domain of CHD4

As previously mentioned, the N‐terminal region of CHD4 (Residues 1–354) comprises two intrinsically disordered regions (IDRs 1 and 2), a HMG domain, and an APR. This region plays a critical role in chromatin remodeling (Figure 1).

In this study, in the IDR1, a rare inherited missense mutation c.22C > T; p.P8S was identified in one MMA case with unknown inherited pattern [29] (Table S1), showing several cardiovascular anomalies: hypertension, left ventricular hypertrophy, and coronary artery disease (Figure 2). The amino acid sequence alignment showed that the mutation was highly conserved across different vertebrate species (data not shown); this residue has been found mutated in human cancer (see Table S1), and it is classified in ClinVar as likely pathogenic (Table 1). Different *in silico* prediction tools yielded conflicting interpretations for this variant. PolyPhen‐2 predicted it as likely pathogenic (score: 0.91), whereas HOPE, AlphaMissense, classified it as benign, and REVEL as undetermined (Table 1). According to HOPE, the variant lies within a disordered region (IDR1) (Table S3), suggesting that substituting proline with serine could alter the local structure. However, the MetaRNN score is low (0.24), indicating limited support for a damaging effect despite the potential structural disturbance and possible impact on CHD4′s remodeling activity. Based on REVEL scores, ACMG guidelines, and all available evidence, the current data are insufficient for a definitive classification, and the variant remains a VUS pending further functional studies.

Furthermore, several authors have described one *de novo* missense mutation, M202I in a cohort of CHD subjects recruited to the Congenital Heart Disease Network Study of the Pediatric Cardiac Genomics Consortium [26, 28]. The proband carrying this mutation presented conotruncal defects including membranous ventricular septal defects (VSD) with extracardiac anomalies [26, 28] (Table S1). Furthermore, this mutation has been also described in individuals with SIHIWES with heart defects [20]. Comparative sequence analysis shows that this mutation is located in a highly evolutionarily conserved region, the HMG domain (Figure 1) [20, 44], attracting the interest of the scientific community to answer if the mutation leads to cardiac disease. *In silico* approaches using PROVEAN and SNAP2, predict that the mutation has a damaging impact with a possible effect in functionality [68]. Recently, to investigate the mechanism by which CHD4‐M202I contributes to cardiac pathology, Shi et al. [26] generated a humanized mouse model of this variant using the CRISPR/CAS9 gene‐editing system; the equivalent mutation corresponds to Chd4‐M195I (Figure 3). Mice with this mutation have developed biventricular hypertrabeculation and ventricular noncompaction (VNC), dying at birth. The authors using different molecular approaches show a significantly increase in proliferation of cardiomyocytes and a concomitant excessive trabeculation with accumulation of ECM proteins and a reduction of one specific ECM protease (ADAM metallopeptidase with thrombospondin Type 1 motif 1 [ADAMTS1]). This phenotype was rescued by administration of ADAMTS1, suggesting that Chd4‐M195I protein has more affinity to endocardial BRG1 (SWI/SNF–related, matrix‐associated, actin‐dependent regulator of chromatin, Subfamily A, Member 4) that cause the failure of derepression of *Adamts1* transcription such that ADAMTS1‐mediated trabeculation termination. In agreement, ACMG classification and *in silico* predictions performed in our study suggest that this variant could be classified as pathogenic (Table 1), given a clear genotype–phenotype correlation.

Outside the HMG‐domain, the inherited variant c.856C > G/p.P286A was identify as heterozygous missense *CHD4* variant in three children with IP with sinus arrhythmias, premature atrial contraction, and mid aortic valve regurgitation ([31]; Table S1, Figure 2). The amino acid sequence alignment showed that the mutation was highly conserved across different vertebrate species (Table S3), and using different *in silico* prediction tools, the mutation was predicted to be benign (e.g., Polyphen2: 0.02 benign; Table 1). The molecular effect of this variant has been analyzed by Liu et al. [31], using SWISS‐MODEL. At Residue P286, no hydrogen bond with the surrounding residues was formed in both wild‐type and the variant residue A286 [31]. In agreement with previous predictions, in this study, the use of HOPE software suggests that the change of proline by alanine could disturb the local structure, though the MetaRNN score is low, suggesting the mutation may be benign (Tables S2 and S3). In concordance, P286A presented a low‐allele frequency (4.09 × 10^−6^ in general population and 5.567 × 10^−5^ in East Asia population) in gnomAD database, but it was not present in the control populations of gnomAD. Considering all the information, the proposed ACMG classification is a VUS mutation.

### 3.10. Genetic Variants in the PHD2 Domain of CHD4

As previously stated, PHD1 and PHD2 (Residues 442–482) are involved in recognizing and interacting with histone modifications, such as those on histone H3 (Figure 1).

Several authors have described a *de novo* missense mutation C467Y that has been related with CHD (tetralogy of Fallot), and intellectual developmental delay [18, 20, 63, 64] (Figure 2). Although the effect on functionality of CHD4 of most of the DNMs remain to be clarified, it is known that the single nucleotide substitution in PHD2 domain (c.4018C > T; p.Cys467Tyr) [18–20], has also been mapped *in silico* in the PHD2 of CHD4 in order to predict its effect. According to Farnung et al. [23], it seems to disrupt the coordination of a zinc ion (Zn^2+^) in the zinc finger of the PHD2, rendering decreased ATP hydrolysis and nucleosome remodeling activity [43]. Furthermore, in our analysis according to HOPE software, the amino acid substitution is predicted to alter the three‐dimensional conformation of the protein, potentially compromising its DNA‐binding capacity. The differences in size and hydrophobicity between the wild‐type cysteine and mutant tyrosine residue are likely to disturb the formation of a hydrogen bond with serine at Position 469 (Table S3). This data agrees with *in silico* predictions showing that the mutation is probably damaging and pathogenic (Polyphen2 = 1; MetaRNN score = 0.98, and REVEL = PP3 strong) (Tables 1, S2, and S3); and in this study has been classified as pathogenic according to ACMG.

### 3.11. Genetic Variants in the CHROMO Domain of CHD4

As previously mentioned, in CHD4, the CDs are duplicated and are located between amino acids 522 and 676 (Figure 1). This highly conserved domain has been found in a large array of organisms, from protists to mammals. The CDs are involved in the binding to the nucleosomes, which contributes to the formation of heterochromatin and gene regulation [55–57]. Therefore, it is an important region of the protein with a key role in nucleosome binding and mobilization, leading to the formation of heterochromatin and gene regulation (Figure 1).

Mutations in these regulatory regions (PHD and CHD) have been proposed to act in a dominant manner because they are located in regions essential to regulate the repression activity of CHD4 in the heart [22]. According to published data, during embryogenesis, Chd4 forms temporally and spatially regulated complexes with several cardiac transcription factors (e.g., Gata4, Nkx2‐5, and Tbx5). This suggests that *Chd4* mutations within these domains could lead to cardiovascular‐specific anomalies [21–23, 43, 59, 70–72].

Several authors have reported mutations in this domain in patients with neurodevelopmental disorders and schizophrenia. However, because it is unknown whether these patients also have heart anomalies, they are not discussed here [64, 73, 74]. Furthermore, Pinard et al. [29] described a missense mutation (T494M) inherited from an unaffected mother in a patient with bilateral angiopathy. *In silico* analyses using HOPE software and PolyPhen‐2 predict that this mutation, located to Chromo Domain 1 (CHD1), may disrupt the 3D structure and impair its DNA‐binding function (Tables S2 and S3). However, our own *in silico* predictions (Tables 1 and S2) suggest that the mutation has only a weak impact, and with the current information, it could be classified as likely benign (Table 1).

In contrast, a novel missense *CHD4* mutation (c.1597A > G/p.K533E) was identified in four unrelated families with childhood IP and sinus arrhythmia (Figure 2). Liu et al. [31] analyzed the molecular effect of this variant using SWISS‐MODEL and found that the original lysine at Position 533 formed four hydrogen bonds with E536, and one hydrogen bond each with G537 and S531. When lysine was replaced by glutamic acid, the hydrogen bonds with S531 and two of the bonds with E536 were lost. An amino acid sequence alignment revealed that this residue is highly conserved across different vertebrate species (Table S3). Despite these findings, various *in silico* prediction tools provided inconclusive results (CADD: 22.8, indicating damaging; REVEL and undetermined; PolyPhen‐2), and the variant supports a likely pathogenic classification according to ACMG (Tables 1, S2, and S3).

A rare splice donor site variant in *CHD4* (c.1686 + 1G > T; see Table S1) was confirmed in six cases of MMA, a cerebrovascular disease characterized by occlusion of large arteries that leads to strokes starting in childhood. This variant is associated with aberrant splicing of exon 11, resulting in a frameshift and subsequent nonsense‐mediated decay [29]. Based on the NMD pathway, we speculate that the RNA harboring this splicing mutation is degraded, leading to low expression of the CHD4 protein or, if expressed, the generation of a truncated protein. CHD4 is highly intolerant to loss‐of‐function variants (*p* *L* *I* = 1) in the general population, and this variant is absent in gnomAD exomes v4.1. Consistent with these findings, predictions from our study using VEP indicate that it is a high‐impact splice donor variant (rs1948532502; ClinVar: likely pathogenic).

### 3.12. Genetic Variants in the ATPase Motor Domain of CHD4

As indicated earlier, the ATPase domain (Residues 731–1192) is critical for chromosomal maintenance and stability, ATP binding, and other functions (Figure 1). Several studies have identified genetic variants in this domain that lead to severe consequences in patients with syndromes such as SIHIWES [18–20, 75, 76] and various cancers [77–79]. The ATPase motor domain is one of the most conserved regions across species, meaning that even a single amino acid change may impair protein functionality. Notably, we mapped 20 rare DNMs onto this domain of CHD4 (Figure 2). Of these mutations, 18 are missense, one is a small deletion, and one is a small duplication. All have been reported in patients with SIHIWES syndrome, presenting congenital heart defects (CHDs), and developmental disorders associated with heart anomalies such as atrial septal defect, ventricular septal defect, pulmonary stenosis, and tetralogy of Fallot (Figure 2, Table S1). Some variants have also been observed in patients with bilateral MMA [29].

According to ACMG classification performed in this study these 18 missense variants are likely pathogenic or pathogenic, suggesting that these mutations are likely deleterious to protein function (Tables 1 and S1). Furthermore, the molecular effects of these mutations have been evaluated using HOPE software, which predicts that most of the mutations could alter the protein′s structure (Table S3).

Additional evidence supports the pathogenicity of the variant R1068H. Farnung et al. [23] showed that residue Arg1068 forms a hydrogen‐bond network with the side chain of Thr1137 and the main‐chain carbonyls of Phe1112 and Gln1119. The R1068H mutation disrupts this network, potentially compromising the integrity of the ATPase fold. Similarly, Trp1148, which is part of ATPase motif Va, contacts the guide strand in a manner similar to Chd1 and Snf2 [23, 31]. Mutation of this residue uncouples ATP hydrolysis and chromatin remodeling [31]. Consequently, the variant R1068H has been classified as pathogenic.

Notably, two variants cluster within the ATPase motor domain, between the ATPase and Helicase domains of CHD4: one small duplication (L1009_V1011dup) and one small deletion (C1012del). Both variants were identified in patients with SIHIWES and CHD presenting serious cardiac defects, such as tetralogy of Fallot (Figure 2; Table S1). *In silico* predictions using VEP indicate that L1009_V1011dup is an in‐frame insertion that duplicates the residues LNV, whereas C1012del results in the deletion of these amino acids (p.Leu1009_Val1011del) without causing a frameshift. In both cases, the predictions suggest a moderate impact, meaning the mutations might alter protein function but are less likely to cause a complete loss of function compared with frameshift or nonsense mutations. Experimental validation through functional assays is necessary to determine their true impact on protein activity. To further support an association between rare *CHD4* variants and cardiovascular anomalies, all mutations described here were absent in both the gnomAD exomeV4.1 and TOPMed databases (Table S2).

### 3.13. Genetic Variants in the C‐Terminal Domain of CHD4

As mentioned, the C‐terminal domain of CHD4 (Residues 1230–1912) is organized in two domains CTD1 and CTD2. CTD1 domain is separated into C1a (Residues 1230–1380) and C1b (Residues (1400–1540), whereas CTD2 domain is formed of C2a (Residues 1700–1800) and C2b (Residues 1800–1912). The C‐terminal domain is implicated in nucleosomal DNA binding activity and in the interaction of CHD4 with the NuRD complex (Figure 1).

We mapped nine rare DNMs to this CHD4 domain (Figure 2), all of which are missense. Four have been reported in patients with SIHIWES syndrome and CHDs, including atrial septal defects; two of these individuals also presented with sinus arrhythmias and premature atrial contractions [31]. Another mutation was recently identified in a patient with dextrocardia [65], and the remaining three were associated with cerebrovascular disease and hypertension [29] (Figure 2, Table S1).

Using ACMG classification, we attempted to predict the effects of these mutations as thoroughly as possible (Tables 1, S2, and S3). As a result, five mutations are classified as likely pathogenic (Y1249D, Y1345D, R1419H, V1608I, and E1646K; Table 1) and four as VUS (D1659E, I1741V, Y1758C, and P1880; Table 1). The Y1249D and Y1345D mutations affect residues within the C1a domain (1230–1380), a key region with an autoinhibitory role in CHD4. This domain regulates protein activity by reducing substrate binding affinity and restricting conformational changes in the translocase domain. *In silico* prediction (HOPE software; Table S3) analyses suggest that replacing tyrosine—a highly conserved and functionally important residue—with aspartic acid has significant detrimental effects. In its native state, tyrosine plays a critical structural role by forming stable interactions through hydrogen bonding and aromatic stacking. Replacing it with aspartic acid introduces a negatively charged residue into a region that relies on structural stability for its autoinhibitory function. This substitution may disrupt the C1a domain′s interaction with the translocase domain, impairing its regulatory role. Moreover, aspartic acid is smaller and has different chemical properties compared to tyrosine, which leads to local destabilization due to the loss of key interactions. The introduction of a negative charge may also repel nearby residues or ligands with similar charges, creating electrostatic repulsion in a region critical for conformational coupling. The affected tyrosine residue is highly conserved across homologous sequences, underscoring its functional importance, whereas the mutant residue (aspartic acid) or similar residues have not been observed at these positions in homologous sequences. This further supports the notion that these mutations are detrimental to CHD4 function. In short, the Y1249D and Y1345D mutations are predicted to compromise the C1a domain′s autoinhibitory function (Figure 1), leading to reduced regulation of substrate binding and remodeling activity of the translocase domain, and ultimately affecting the overall function of CHD4. In agreement with these findings, *in silico* predictions with REVEL (Table 1) classify these mutations as PP3‐moderate. Further, 3D‐simulation and functional studies are required to describe the clinical significance of this mutation.

The R1419H, V1608I, E1646K, and D1659E mutations are located within the SLIDE domain—a key component of the bipartite SANT‐SLIDE structure (C1: Residues 1200–1539 and C2: Residues 1700–1912)—which facilitates CHD4′s interaction with nucleosomal DNA, particularly across the disorder linker region (Residues 1540–1699) (Figure 2). This domain is essential for chromatin remodeling, as it binds double‐stranded DNA through a flexible, electrostatically charged surface.

Among these variants, R1419H shows consistent classification across sources: both ClinVar and ACMG criteria identify it as likely pathogenic, in agreement with previous reports [65]. In contrast, E1646K and D1659E had no available ClinVar entries, and our integrated analysis allowed their classifications as likely pathogenic and VUS, respectively. The V1608I variant, previously listed in ClinVar with a conflicting classification (CS), was evaluated in this study and classified as likely pathogenic based on the available information available (Tables 1, S2, and and S3). A possible explanation is that V1608I involves substituting valine with isoleucine, two residues with similar hydrophobic properties and side‐chain sizes, which likely has minimal impact on structural stability or DNA‐binding affinity. In the case of E1646K, the mutation replaces a negatively charged glutamic acid with a positively charged lysine. Although this alters the local electrostatics, it does not seem to disrupt the balance necessary for DNA binding. D1659E represents a conservative substitution between two negatively charged residues (aspartic acid and glutamic acid) and is unlikely to significantly affect the structure or function due to their comparable properties. Overall, while these variants occur within a region crucial for DNA interaction, their computational predictions and structural context suggest a spectrum of potential impact, from likely benign or neutral (D1659E) to possibly destabilizing (V1608I and E1646K). Nonetheless, experimental validation will be essential to confirm the functional consequences of these substitutions within the SANT‐SLIDE domain.

Three mutations; I1741V, Y1758C, and P1880S, are located in the CTD2 domain, which is comprised of the subdomains C2a (Residues 1700–1800) and C2b (Residues 1800–1912) (Figure 2). These mutations have been reported in patients with bilateral MMA who suffer from cerebrovascular disease, hypertension, and coronary artery disease (Table S1, Figure 2). Specifically, I1741V is located within the unique APR region (Residues 1735–1742, sequence YWLLAGII) and may impair interactions with the NuRD and ChAHP complexes. Pinard et al. [29] noted that this mutation occurs in a region required for interaction with PCNT (pericentrin), suggesting that the variant could disturb this interaction and its associated function. In our study, ACMG classification describes this mutation as VUS in contrast with ClinVar that classifies it as likely pathogenic, therefore more studies are needed to define the clinical significance of this mutation.

The other two mutations, Y1758C and P1880S, have uncertain pathogenic classifications in ClinVar. Although *in silico* predictions suggest that both may be deleterious (Tables 1 and S2), they remain classified as variants VUS according to ACMG criteria. At the molecular level, the Y1758C mutation is situated within a critical region for PCNT interaction. In the wild‐type protein, the tyrosine residue at Position 1758 forms a stabilizing hydrogen bond with alanine at Position 1739, which contributes to the local structural integrity of this region. Replacement of tyrosine with cysteine disrupts this hydrogen bond due to differences in size‐chain, polarity and hydrophobicity, leading to local destabilization that may impair protein–protein interactions. Additionally, the wild‐type tyrosine is not easily substituted, as cysteine is not typically observed at this position in homologous sequences, further supporting the idea that this substitution is detrimental.

Similarly, the P1880S occurs in a critical region required for PCNT interaction. The wild‐type residue—proline—is known for its rigidity due to its cyclic structure, which imposes a specific backbone conformation essential for maintaining structural integrity. Replacing proline with serine likely disrupts this rigidity, altering the local backbone conformation and destabilizing the region. Conservation analysis further supports the functional importance of this residue, as proline is uniquely conserved at this position among homologous CHD4 sequences. Based on these structural and conservation insights, the P1880S mutation is likely damaging and may impair CHD4 function. Although these findings suggest a potentially damaging role, additional functional and segregation studies are required to establish the clinical significance of these variants.

## 4. Conclusion

The comprehensive analysis of CHD4′s structure and function reveal that it is a multifunctional protein organized into five major regions: the N‐terminal region, the PHD, the CD, the ATPase motor, and the C‐terminal region (Figure 1). Each region plays a unique role in CHD4′s biological activity, underscoring the importance of integrating both experimental and computational approaches to fully understand its functional complexity.

Building on compiled data and the classification of previously described human *CHD4* variants linked to rare pathological phenotypes, we focused on three conditions: SIHIWES, MMA, and childhood IP with sinus arrhythmia. Across these conditions, we identified a total of 33 missense variants, 1 splicing variant, 1 in‐frame deletion, and 1 in‐frame insertion in patients exhibiting cardiovascular anomalies.

The output of classification according to ACMG guidelines and subsequent refinements allow us to classify seven variants as pathogenic, 19 as likely pathogenic, one as likely benign, and six as uncertain significance. This classification is highly valuable to stratify variants for additional functional studies and for predicting the phenotypic outcomes, particularly as all patients in our study present various heart and cardiovascular anomalies. The data provided herein serves as a critical resource for the accurate interpretation of *CHD4* variants in the context of diagnosing rare diseases associated with cardiovascular defects.

Ultimately, our findings emphasize the need for further experimental validation and functional assays to elucidate the precise mechanisms by which *CHD4* mutations contribute to disease. This, in turn, will enhance genetic diagnostics and precision medicine approaches for affected patients, paving the way for improved clinical outcomes in the management of rare cardiovascular disorders.

## Ethics Statement

The authors have nothing to report.

## Conflicts of Interest

The authors declare no conflicts of interest.

## Author Contributions

A.N.: conceptualization; formal analysis; project administration; resources; investigation; validation; supervision; visualization; writing—original draft; writing—review and editing. P.G.A.: conceptualization; formal analysis; funding‐acquisition; project administration; resources; investigation; validation; supervision; visualization; writing—original draft; writing—review and editing. M.Y.: investigation; methodology; writing—review and editing. R.B.: investigation; methodology; writing—review and editing. A.M.H‐E. and F.E.A‐S.: conceptualization; resources; writing—review and editing.

## Funding

This study was supported by Ministerio de Ciencia e Innovación (PID2020‐114773GB‐I00 from MCIN/AEI/10.13039/501100011033).

## Supporting Information

Additional supporting information can be found online in the Supporting Information section.

## Supporting information


**Supporting Information 1** Table S1: Genotypes and phenotypes associated with the 36 *CHD4* mutations analyzed in this study, all identified in patients with cardiovascular anomalies. References for each mutation are provided. Information on whether the corresponding wild‐type residue is mutated in cancer was obtained from COSMIC, coded as follows: ++ (the residue is mutated and the specific variant reported here has been observed in cancer), + (the residue is mutated in cancer but this specific variant has not been reported), – (the residue is not mutated in cancer).

**Supporting Information 2** Table S2: Detailed information on the 36 *CHD4* mutations described in this study. The table reports variant characteristics (mutation, type, and domain), predicted biochemical impact (ATPase hydrolysis and nucleosome remodeling), supporting experimental findings, multiple computational prediction scores (MetaRNN, PolyPhen‐2, AlphaFold structural analysis, REVEL, and REVEL‐score), ACMG classification with the criteria used, bibliographic references, additional relevant notes, and population allele frequencies from gnomAD v4.1 and TOPMed datasets. References are included for each entry. ND, not data; VUS, variant of uncertain significance.

**Supporting Information 3** Table S3: Project HOPE predictions for the 33 missense *CHD4* variants examined in this study. For each variant, changes in amino acid properties are indicated as + (a change is detected at that position) or – (no change detected), across three features: amino acid size, hydrophobicity, and charge. Evolutionary conservation is shown as + (the mutant residue is not observed at the wild‐type position in homologs) or – (the mutant residue is observed at that position). Structure prediction results, MetaRNN scores, and the predicted molecular effect are also provided for each variant.

## Data Availability

Data sharing is not applicable to this article as no datasets were generated or analyzed during the current study.
